# Survival Bias and Crosstalk between Chronological and Behavioral Age: Age- and Genotype-Sensitivity Tests Define Behavioral Signatures in Middle-Aged, Old, and Long-Lived Mice with Normal and AD-Associated Aging

**DOI:** 10.3390/biomedicines9060636

**Published:** 2021-06-02

**Authors:** Lydia Giménez-Llort, Daniela Marin-Pardo, Paula Marazuela, Mar Hernández-Guillamón

**Affiliations:** 1 Institut de Neurociències, Universitat Autònoma de Barcelona, E-08193 Barcelona, Spain; daniela.marin@e-campus.uab.cat; 2 Department of Psychiatry and Forensic Medicine, School of Medicine, Universitat Autònoma de Barcelona, E-08193 Barcelona, Spain; 3 Vall d’Hebron Research Institute (VHIR), E-08035 Barcelona, Spain; paula.marazuela@vhir.org (P.M.); mar.hernandez.guillamon@vhir.org (M.H.-G.)

**Keywords:** survival, aging, Alzheimer’s disease, heterogeneity, long-life, gait analysis, cognition, BPSD

## Abstract

New evidence refers to a high degree of heterogeneity in normal but also Alzheimer’s disease (AD) clinical and temporal patterns, increased mortality, and the need to find specific end-of-life prognosticators. This heterogeneity is scarcely explored in very old male AD mice models due to their reduced survival. In the present work, using 915 (432 APP23 and 483 C57BL/6 littermates) mice, we confirmed the better survival curves in male than female APP23 mice and respective wildtypes, providing the chance to characterize behavioral signatures in middle-aged, old, and long-lived male animals. The sensitivity of a battery of seven paradigms for comprehensive screening of motor (activity and gait analysis), neuropsychiatric and cognitive symptoms was analyzed using a cohort of 56 animals, composed of 12-, 18- and 24-month-old male APP23 mice and wildtype littermates. Most variables analyzed detected age-related differences. However, variables related to coping with stress, thigmotaxis, frailty, gait, and poor cognition better discriminated the behavioral phenotype of male APP23 mice through the three old ages compared with controls. Most importantly, non-linear age- and genotype-dependent behavioral signatures were found in long-lived animals, suggesting crosstalk between chronological and biological/behavioral ages useful to study underlying mechanisms and distinct compensations through physiological and AD-associated aging.

## 1. Introduction

Success in aging and increased life expectancy are historical achievements of the last century. However, the fast rate of social aging and age-related chronic diseases urge a bio-psycho-social safety net able to hamper the global burden that it is projected to happen in the next decades [1,2,3]. In addition, the individual differences in the complex process of aging leads to old age being the most heterogeneous period of life, thus demanding multidisciplinary gerontology and geriatric approaches [4]. Concerning mental health, World Health Organization (WHO)’s last report warns that the prevalence of psychiatric and neurological disorders in older adults, which already account for 6.6% of the total disability-adjusted life years (DALYs), will also increase [5]. Foremost, these disorders are likely to happen in an already complex multimorbid scenario that in most cases will include frailty and age-related medical conditions, strongly affecting the quality of life of old people and their caregivers. In this worrisome forecast, prevalence and correlates of psychiatric disorders among nursing home residents without dementia are the topic of interest of the systematic review and meta-analysis [6].

In this context, the syndromic nature of Alzheimer’s disease (AD), the most common neurodegenerative disease representing more than 80% of the cases of dementia worldwide in older people, exemplifies the challenges of such a complex scenario [7]. Thus, besides progressive loss of judgment, memory, and high functions as clinical hallmarks of AD, a wide array of neuropsychiatric symptoms (NPS), also referred to as ‘Behavioral and Psychological Symptoms of Dementia (BPSD)’ including behavioral and daily life activity impairments, exacerbate the functional impairment of patients and the burden of disease [8,9]. They are present in 90% of patients as the disease progresses, with noteworthy prevalence, such as the 20–80% for agitation; 9–63% for delusion; 11–46% for aggression, 4–41% for hallucinations [10]. BSPD are also considered the core symptoms of different dementia subtypes from early on in the case of dementia, with Lewy bodies or its behavioral variant frontotemporal dementia, and is thus becoming essential for diagnosis [11]. Principal component analysis condensed cognitive/behavioral variables into seven factors, namely: general-cognitive, constructional abilities, hyperactivity, psychosis, anxiety, mood-excitement and mood-depression/apathy, concluding that cognition and other behavioral aspects are independent dimensions [12,13]. Clinical management of BPSD is challenging, requesting the support of non-pharmacological interventions [14,15], mostly in the case of antipsychotics since they have been associated with an increased mortality risk [10,11,15,16] as was also demonstrated in animal models [17]. Due to this complexity and heterogeneity in the human manifestation of AD neuropathology, modeling the whole array of cognitive and behavioral deficits in animal models has been a challenge during the last two decades [18]. At the biological level, the neurobiological basis for the cognitive and behavioral heterogeneity in Alzheimer’s disease reflects such a complexity [7]. Thus, besides synaptic dysfunction, beta-amyloid, and tau neuropathological hallmarks of Alzheimer’s disease, other key pathological factors such as brain oxidative stress, neuroinflammation, neurovascular dysfunction, and neuroimmunoendocrine crosstalk have been associated with it [19,20].

Recently, new evidence refers to a high degree of heterogeneity in the patterns and temporal progression of clinical symptoms in Alzheimer’s disease, indicating several subgroups of patients [21]. At the translational level, this heterogeneity has also been demonstrated in animal models for the disease [22,23,24]. On the other hand, new perspectives on psychological science also point at emerging data supporting the default-executive coupling hypothesis of aging, where gains on cognitive aging fill the gap that results from a framing effect and focus on losses in cognitive ability as hallmarks of aging [25,26]. In this regard, experimental gerontologists highlight the relevance of using aged animals to mimic the complexity of the physiological and multifactorial pathological aging processes in humans [27,28]. This is most important when studying the age and disease interaction effects in the expression of phenotypic characteristics of late-onset neurodegenerative processes and the screening for drug discovery [18,29]. However, the number of research works using old (+18 months of age) and very old (+21 months of age) or naturally long-lived animals have to overcome the sparsity of studies [30,31,32], to confront methodological difficulties due to the natural constraints of aged animals [33,34] and severe limitations due to mortality bias [35,36]. Since BPSD are highly prevalent also in old people without dementia [6], translational research using AD-models can also provide the benefit of the observation of the age-matched old wildtype specimens as a source of knowledge of the normal aging process, as is presented here.

Compared with aged control populations, dementia is associated with increased mortality [37,38], and this vulnerability can be enhanced in some AD patients when atypical antipsychotics are used for the management of their BPSD [10,14,15,16]. In this context, the heterogeneity in the progress of clinical symptoms in the older AD patients is considered a problem for public health planning that urgently needs prognostic indicators specific to this aged population [21,39]. Among them, comorbidity, functional disability, and demographic characteristics such as older age, male gender, and low education are identified as those increasing the risk of death [37,40,41]. Similarly, despite the females of several animal models of AD exhibiting a worse neuropathological status than males [42,43], increased mortality rates in males has been reported in many of them [35,44,45], and is related to an impaired neuroimmunoendocrine system [19,35,46,47]. At the experimental level, this reduced survival in males can result in a mortality bias in studies using old and very old animals (by standard, 18 and 24 months of age, respectively), where male subjects with worse life prognostics cannot be included because they are already dead [22,23,48].

Interestingly, in the APP23 model [49], we have observed that male mice harboring APP23 mutations can reach old and very old ages and can therefore be a useful model to elucidate unbiased age-dependent disease features in the male sex. Therefore, in the present project, we first confirmed long-lived survival curves in male APP23 compared to APP23 females and respective control wildtype (WT) mice that were grown and aged under the same living conditions. Then, three cohorts of 12, 18, and 24 months of age male APP23 mice were successively evaluated in a battery of seven behavioral tests [18] for the comprehensive screening of exploratory activity and gait analysis, cognitive and NPS-like symptoms, as compared to their WT counterparts, the gold-standard C57BL/6 mice strain, with normal aging.

## 2. Materials and Methods

### 2.1. Animals

APP23 transgenic male mice (Hemizygote B6, D2-TgN[Thy-APP23]-23-Tg mice, Novartis, Basel, Switzerland) [49] overexpress the mutant human-type APP protein with the Swedish mutation (K670M/N671L) under the control of the murine brain and neuron-specific Thy-1 promoter (thymocyte antigen-1). Hemizygous APP23 mice were backcrossed with the wildtype (WT) C57BL/6 mice (Janvier Labs, Le Genest-Saint-Isle, France), and the APP genotype was tested by Transnetyx (Cordova, TN, USA). The APP23 mice and their wild-type littermates were aged in the Rodent Platform—Lab Animal Service (LAS), the Vall d’Hebron Research Institute animal facility to obtain the final study cohort. Animals were maintained under standard laboratory conditions of food (SAFE A004, Panlab, S.L., Barcelona, Spain) and water ad libitum, 22 ± 2 °C, a 12-h dark/light cycle, 50–60% humidity.

### 2.2. Experimental Design

The experimental design was defined in three steps. First, survival curves were calculated based on the two colonies of 432 APP23 mice and 483 WT C57BL/6 mice. After that, behavioral assessments were performed in 58 male mice, 29 APP23 mice and their 29 WT C57BL/6 mice littermates. Three sets of animals of 12, 18, and 24 months of age, were transferred to Servei d’Estabulari UAB, Universitat Autònoma de Barcelona, which is located at a distance of 17.4 km from Vall d’Hebron Research Institute (a 15 min ride). All the animals were housed three to four per cage and maintained (Makrolon, 35 × 35 × 25 cm^3^) under standard laboratory conditions (12 h light/dark, cycle starting at 8:00 h, food and water available ad libitum, 22 ± 2 °C, 50–60% humidity). One week after arrival, the animals were successively assessed using a battery of seven tests to evaluate four behavioral and functional dimensions: sensorimotor, cognitive, emotional, and daily life activities. All procedures followed Spanish legislation on ‘Protection of Animals Used for Experimental and Other Scientific Purposes’ and the EU Council directive (2010/63/EU) on this subject. The protocol CEEAH 3588/DMAH 9452 was approved the 8th of March 2019 by Departament de Medi Ambient i Habitatge, Generalitat de Catalunya. The study complies with the ARRIVE guidelines developed by the NC3Rs and aims to reduce the number of animals used [50].

### 2.3. Behavioral Assessments

Behavioral assessments [18] were performed from 9:00 h to 13:00 h, in a counterbalanced manner and blind to the experiment. Assessments were performed blind to the experiment in a counterbalanced manner. The experimental groups were the following: 12m-WT (*n* = 10), 12m-APP23 (*n* = 9), 18m-WT (*n* = 10), 18m-APP23 (*n* = 11), 24m-WT (*n* = 9), and 24m-APP23 (*n* = 9).

#### 2.3.1. Day 1. Corner and Open-Field Tests (CT and OF)

Animals were individually placed in the center of a clean standard home cage, filled with wood shave bedding. Neophobia was evaluated in the corner test (CT) for 30 s through the number of corners visited (CTc), latency to perform the first rearing (CTlatR), and the number of rearings (CTr). Immediately after, mice were placed in the center of an open-field (OF), beige metal drawer (metalwork, 50 × 40 × 25 cm) and were observed for 5 min. The sequence of behavioral events that define the animal’s ethogram was recorded as follows: duration of freezing behavior (LatM, latency to move), latency to leave the central square (LatC) and that of entering the peripheral ring (LatP), as well as latency and total duration of self-grooming behavior (LatG and tG, respectively). Horizontal (crossings of 10 × 10 squares) and vertical (rearings with wall support) locomotor activities were also measured. Bizarre behaviors observed in this test (stereotyped stretching, stereotyped rearing, backward movements, and jumps) were also measured according to previously reported criterion [51]. During the tests, defecation boli and urination were also recorded.

In the present work, we propose gait analysis of locomotion in the open field to measure the number of forward locomotion episodes (walking preceded and followed by a rest) and the number of crossings covered on each, as complementary to measures to horizontal and vertical activity time courses [18] and classical total counts.

#### 2.3.2. Day 2. Recognition Tests (OF2 and OR)

The day after, the animals were retested in the open-field (OF2) to evaluate the behavioral response when they confronted the same anxiogenic environment again. Analysis of activity was done during the first minute of the test, the time period where we have previously described AD-genotype differences [52]. Immediately after, animals were moved to a standard home cage, where they remained for one minute before being reintroduced to the field where two objects were now allocated to administer the novel object recognition test. The animals were assessed (4 h apart) for their ability to recognize a familiar object (S, sample) from a new one (N). Each of these sessions consisted of a “sample trial” followed by a “test trial.” In the sample trial, the animals were placed in the open-field (a known environment) and left to explore (nose directed to the object not less than 1 cm) two identical objects, S1 and S2 (glass bottles, 15 × 12 cm, 5 cm diameter), equally spaced in the floor of the apparatus until they reached the criteria of exploration of both for 20 s. The time required to reach the criteria was named “time 1.” One minute later, animals were reintroduced in the apparatus for 5 min (test trial) where two different non-explored objects were located: an identical copy of the sample object (S3) and a completely new object (N, rectangular aluminum can, 15 × 10 cm, 4 cm high). Preference for the new object was measured through the index TN−TS/TN+TS where TS and TN are the time spent exploring “S3” and “N,” respectively.

#### 2.3.3. Day 3. Spontaneous Alternation in the T-Maze (TM-SA)

On the third day, the black T-shaped maze (woodwork) consisted of two short arms of 25 × 8 cm and a long arm of 30 × 8 cm. The animals were placed inside the long arm of the maze with its head facing the end wall. The latencies of the first movement, start walking, arriving at the intersection of the arms, and completing the exploration of the maze were recorded. Finally, defecation boli and urination were also recorded.

#### 2.3.4. Day 4 to 9. Morris Water Maze Test (MWM)

Mice were trained, four trials per day, spaced 30 min apart, to locate a platform (14 cm diameter) in a circular pool for mice (120 cm diameter, 80 cm height, 25 °C opaque water), and were covered with a completely black curtain. Mice were gently released (facing the wall) from one randomly selected starting cardinal point and allowed to swim until they escaped onto the platform. On the first day, a cue task (CUE, DAY 1) assessed the visual perceptual learning and memory of a visible platform, elevated 1 cm above the water level in the N position and indicated by a visible striped flag (5.3 × 8.3 × 15 cm). Extra maze cues were absent in the black curtain. During the next four consecutive days (PT1-PT4, DAY2-DAY5), the mice searched for a hidden platform that was located in the middle of the S quadrant. White geometric figures hung on each wall of the room were used as external visual clues. In all trials, mice failing to find the platform were placed on it for 10 s, the same period as the successful animals. On the last day, 2 h after the last trial of the place-learning task, a probe trial without the platform was administered during 60 s to assess spatial memory for the previously trained platform location.

In all the learning tasks, the variables of time (escape latency), distance, and speed were also recorded by a computerized tracking system (ANY-Maze v. 5.14, Stoelting, Dublin, Ireland). The number of crossings over the removed platform position (annulus crossings), the time spent, the distance traveled in each quadrant, and the swimming speed were also analyzed.

### 2.4. Statistics

Statistical analyses were performed using GraphPad Prism 6 and SPSS Statistics 20.0 software. All data are presented as mean ± SEM or percentage. To evaluate the effects of (S) sex, (G) genotype and (A) age factorial analysis design was applied. Differences were studied through multivariate General Lineal Model analysis (mGLM), followed by post-hoc Duncan’s test comparisons. Differences between (G) genotype and (A) age × (T) time interval interactions in the different MWM tasks were analyzed by Repeated Measures ANOVA (RMA). Cox regression was used for multivariate analysis of survival. In all cases, statistical significance was considered at *p* < 0.05.

## 3. Results

### 3.1. Genotype and Sex-Dependent Increased Mortality Rates

**Figure 1:**
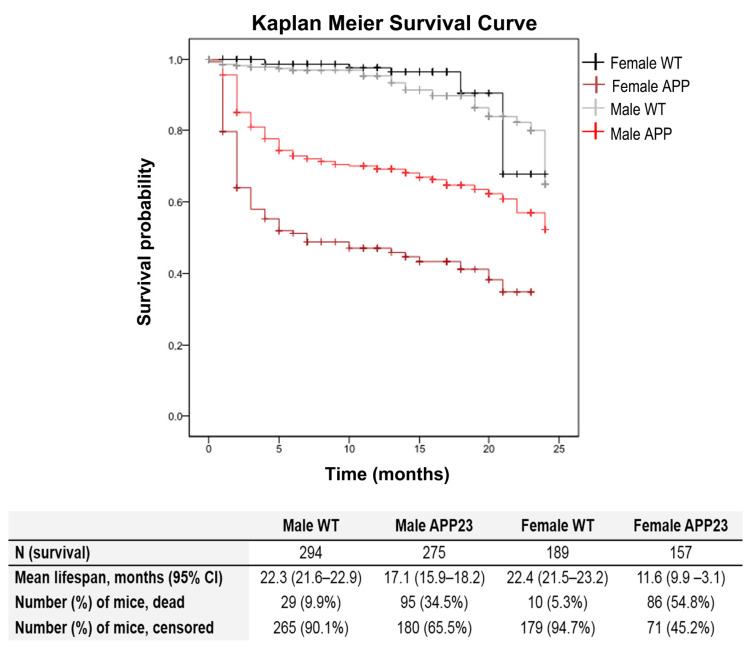
Survival—Effect of age and AD-genotype on survival. Data are expressed by mean and 95% CI, or percentage.

Survival curve (see Figure 1) based on 916 cases and 220 events or natural deaths showed genotype-dependent increased mortality in APP23 mice (G, *** *p* < 0.001), with a severe initial drop of survival, mainly during the first two months of age. Although sex did not influence the risk of mortality in WT littermates, the effect of APP23 genotype in the overall survival was significantly different depending on the sex (S, *** *p* < 0.001). Mortality risk in female APP23 mice was 12.3 times higher than female WT littermates and 2.1 times higher than male littermates with their same AD-genotype. In the case of males, the ratio was 3.6 times higher mortality in the APP23 genotype than male WT littermates.

### 3.2. Strong Contribution of Age and Specificity of Genotype Factor on Key Variables for AD-Related Phenotype

As summarized in Table 1, the effect of age was shown in most behavioral variables (A, *** *p* < 0.001) while genotype differences mainly were related to horizontal and vertical activities, thigmotaxis, and coping with stress strategies. Overall, T-maze resulted in being the most sensitive test to genotype effects.

**Figure 2:**
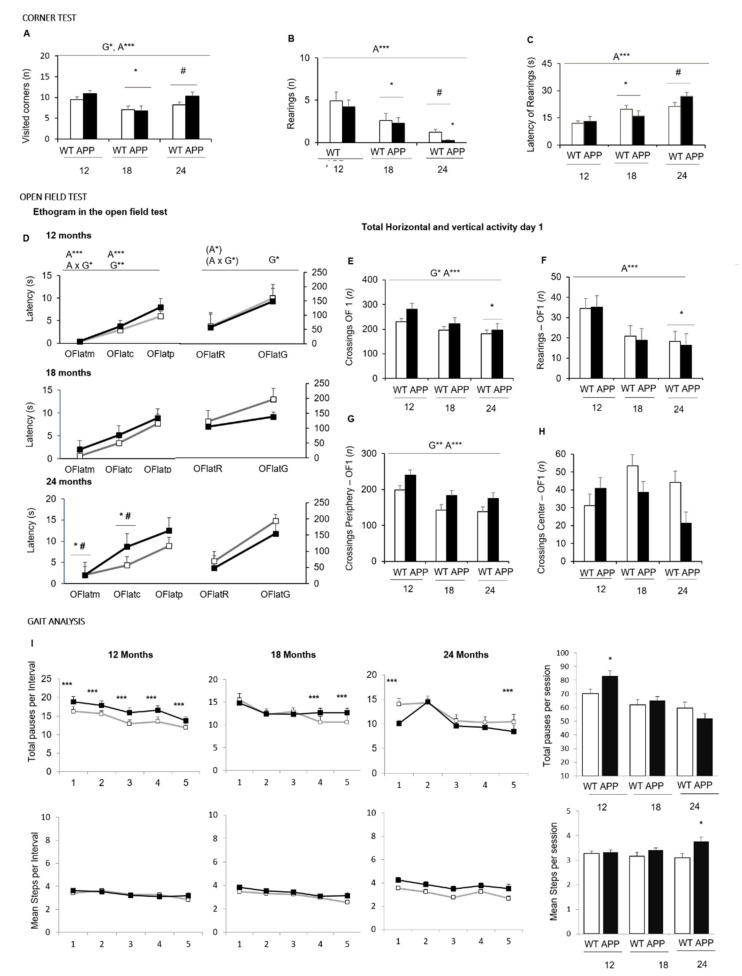
NPS-like behaviors and gait analysis in the corner and open-field test—Effect of age and AD-genotype on the neophobia and exploratory activity elicited in a new home cage (corner test, (**A**–**C**)) and the open-field test (**D**–**I**) at 12, 18, and 24 months of age. Data are expressed by Mean ± SEM. WT: wildtype mice; APP: APP23 mice. Corner test: Visited corners (**A**), number (**B**), and latency of rearings (**C**). Open field test: Ethogram, the temporal sequence of behavioral events in the open field test, Variables: OFlatm, latency of the first movement; OFlatc, latency to leave the central area; OFlatp, latency to enter into the periphery; OFlatR, latency of the first rearing; OFlatG, latencies of the first grooming (**D**). Total horizontal (**E**) and vertical (**F**) activity in the open-field test. Total horizontal activity in the periphery (**G**) and the central area (**H**). Gait analysis: Total number of pauses (**I**) and mean number of crossings in each time interval and in the total time of the open field test. Analysis of variance 2 × 3: Effects of genotype (G), Age (A) * *p <* 0.05, ** *p <* 0.01, *** *p <* 0.001. Post-hoc analysis: * *p <* 0.05 vs. 12 months of age, # *p <* 0.05, vs. 18 months of age; * *p <* 0.05 vs. the corresponding WT control group.

In the corner test, the horizontal locomotor activity, as measured by the number of visited corners (Figure 2A), was higher in APP23 mice as compared to WT animals (G*, *p <* 0.05). However, an age effect pointed at 12 and 24 month animals being more active in comparison with those of 18 months of age (*p* < 0.05, in both cases). In the case of vertical activity, the latency to perform the first rearing (Figure 2C) progressively increased with age (A***, *p <* 0.001). Conversely, the total number of rearings (Figure 2B) recorded decreased as well (A***, *p <* 0.001). Both variables indicated that vertical activity dramatically decreased in the 24-month-old APP23 mice compared to the WT littermates (* *p <* 0.05), unveiling a genotype difference at this age.

The ethogram describing the temporal sequence of behavioral events of animals in the open-field test (Figure 2D) showed that the actions exhibited had a strong effect on age, together with genotype or genotype x age interaction effects. That is, the latency of the first movement (OFlatm) and to leave the central area (OFlatc) were strongly dependent on age (A***, *p <* 0.001 and A**, *p <* 0.01, respectively) but also modulated by genotype (OFlatm, A × G*, *p <* 0.05; OFlatc, *p <* 0.01). The performance of the first vertical exploration in the periphery was modulated by age (A*, *p <* 0.05) and age x genotype interaction (A × G*, *p <* 0.05) with shorter latencies in the APP23 mice as compared to WT littermates due to the presence of stereotyped vertical rearing (without wall support). Self-grooming was affected by genotype with shorter latency.

**Figure 3:**
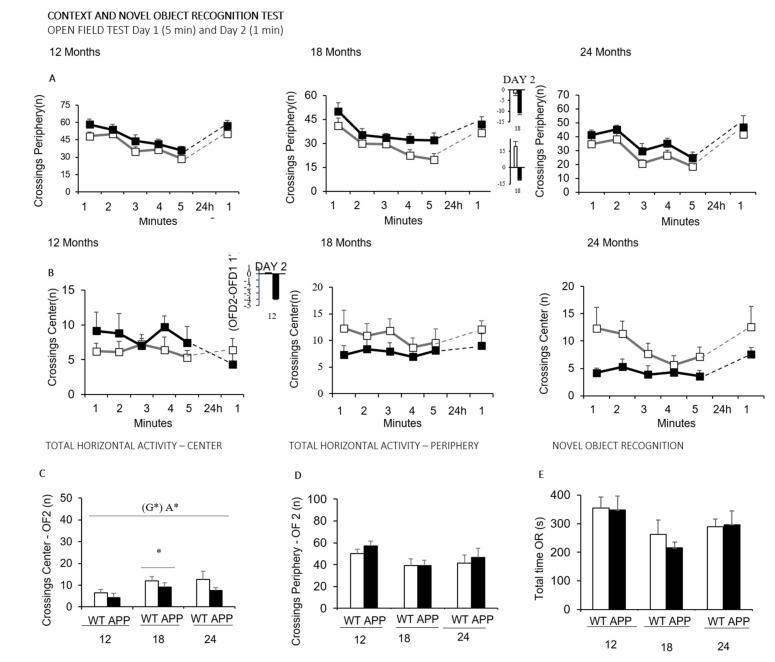
Context and novel object recognition tasks. Data are expressed by mean ± SEM. Time course of the horizontal activity (number of crossings) exhibited in the periphery (**A**) and the center (**B**) of the open-field test on Day 1 and 2, in 12-, 18- and 24-month-old WT (white squares) and APP23 (black squares). (**C**) Total horizontal activity (number of crossings) in the periphery on Day 2, in 12-, 18- and 24-month-old WT (white bars) and APP23 mice (black bars), (**D**) Total horizontal activity (number of crossings) in the center on Day 2, in 12-, 18- and 24-month-old WT (white bars) and APP23 mice (black bars). (**E**) Total time of exploration of the novel object (seconds) in the object recognition test in 12-, 18- and 24-month-old WT (white bars) and APP23 mice (black bars). Analysis of variance 2 × 3: Effects of genotype (G), Age (A), * *p <* 0.05. Post-hoc analysis: * *p* < 0.05 vs. 12 months of age.

The total activity exhibited in the open field test on Day 1 (Figure 2E–H) indicated that the main effects were attributed to age factor (A***, *p* < 0.001) in both the total horizontal (Figure 2E) and vertical (Figure 2F) activities, with a progressive reduction of both variables with aging. Genotype differences were also found in the horizontal activity exhibited on the first day (Figure 2E, G*, *p <* 0.05), with a higher total number of crossing shown by APP23 mice than WT littermate mice. Statistically significant differences were mainly shown at 12 and 24 months of age, and they were due to the activity performed in the periphery (Figure 2G, G**, *p <* 0.05). At 12 months of age, the genotype-dependent increase of activity was observed in both the periphery and the central area, while at the older ages, the APP23 mice showed lower activity in the center as compared to WT, albeit that these differences did not reach the statistical significance until the second day of the test (see Figure 3B; G*, *p <* 0.05; A*, *p <* 0.05).

The increased horizontal activity of APP23 mice compared to WT was further examined through a gait analysis as illustrated in Figure 2I. The performance also showed genotype effects. Thus, at 12 months of age, the increased total activity of APP23 mice did not result from a walking performance covering a greater number of crossings per unit of movement per se, but due to a higher number of walking episodes per interval of time. The total number of episodes was significantly higher as well (G*, *p <* 0.05). With aging, the number of episodes was reduced. At 24 months of age, the variable that evidenced the genotype effects was the number of crossings covered, which was higher in APP23 than their WT littermate group (* *p <* 0.05).

The two recognition tasks performed during the novel object recognition test administered on Day 2 are illustrated in Figure 3. That is, the horizontal activity exhibited when the animals were re-exposed to the open field test (Figure 3A–D) and the discrimination of a novel object in the novel object recognition test (Figure 3E). As shown, when the animals were introduced in the corner of the periphery, both genotypes performed a similar locomotor activity. However, the comparison with their performances on their first experience in the open field test (18 months of age, OFD2-OFD1, minute 1) as well as the before–after activity levels unveiled genotype differences at 12 (Figure 3A) and 18 (Figure 3B) months of age. In the next part, the novel object recognition test showed a low sensitivity to the genotype, only noticed at the oldest age by a lower difference in the latency to explore the novel and familiar objects than WT littermates.

**Figure 4:**
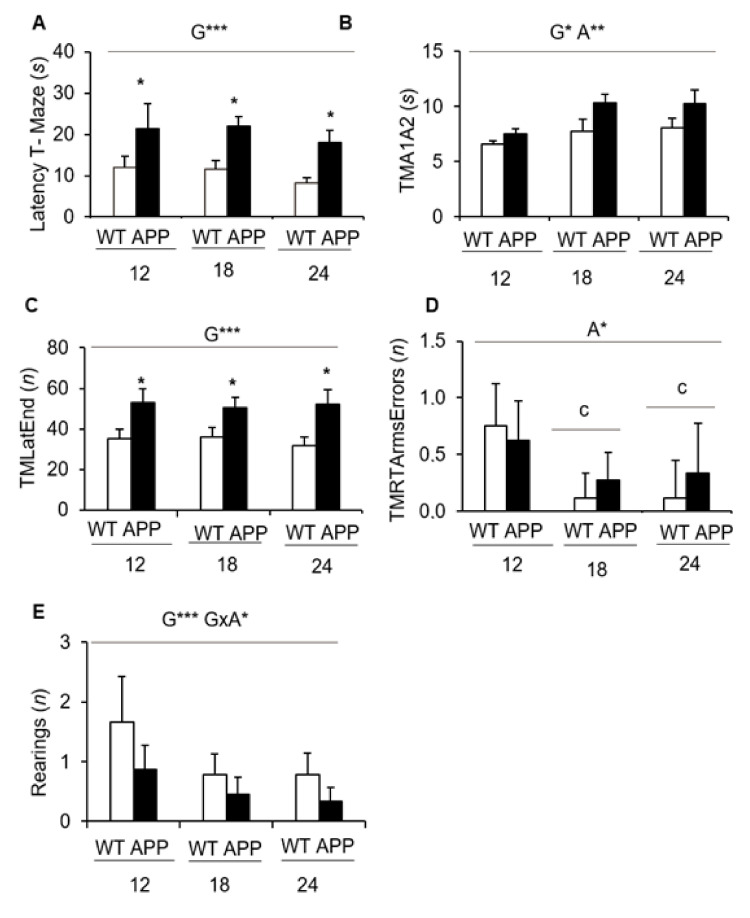
Coping with stress strategies, risk assessment, and spontaneous alternation in the T-Maze. (**A**) Latency to cross the intersection; (**B**) Total time invested in the exploration of the left and right arms; (**C**), Time to complete the exploration of the maze; (**D**), Total number of errors performed during the exploration of the maze; (**E**) Vertical exploratory activity. Data are expressed by means ± SEM. WT: wildtype mice; APP: APP23 mice. Analysis of variance 2 × 2: Effects of genotype (G), age (A), genotype × age (G × A), * *p <* 0.05, ** *p <* 0.01, *** *p <* 0.001. Post-hoc analysis: ^c^ *p* < 0.05 vs. 12 months of age; * *p <* 0.05 vs. the corresponding WT control group.

In the T-maze (Figure 4), at all the ages studied and as compared to WT littermates, the APP23 mice took a longer time to reach the intersection of the vertical arm (not shown) and once there to cross it (Figure 4A, G***, *p <* 0.001). In addition, they also spent more time in both arms (Figure 4B, G*, *p <* 0.05) and required more time to complete the test (Figure 4C, G***, *p <* 0.001). The factor age was also a determinant for the time spent in the arms (Figure 4B, A**, G*, *p <* 0.01). The analysis of errors revisiting the arms that had been already explored indicated a significant age effect (A*, *p <* 0.05) with a decrease at 18 and 24 months of age as compared to 12 months (*p <* 0.05). Furthermore, a decrease in the number of rearings in the APP23 mice, with a genotype x age interaction (G × A*, *p <* 0.05), was found.

To further clarify the results and explain the interrelationships among the various tasks, correlation analyses were performed using data from all the animals in this study (not shown). In the animals’ sensorimotor activity measurements, the most prominent correlation was between the open field activity, more specifically on the crossings in the periphery, and the exploration activity obtained in the test corners (*p <* 0.005). Additionally, there was a correlation between the crosses in the periphery of the open field and the exploration time of the arms of the T-maze (*p <* 0.005). On the other hand, a correlation was obtained between the number of corners visited and the amount of exploratory activity performed on the first day of the open field (*p* < 0.05).

**Figure 5:**
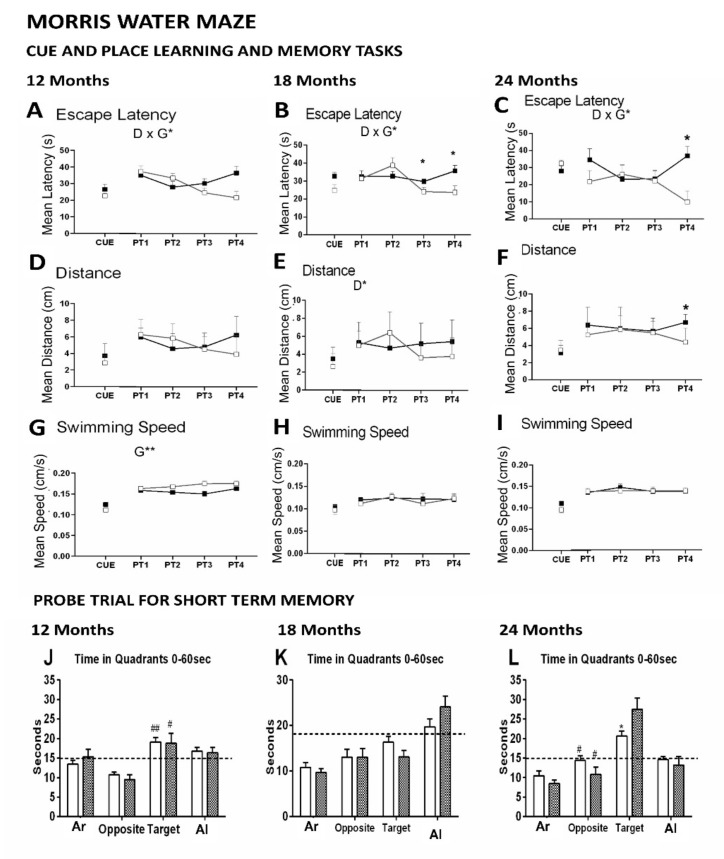
Quantitative analysis of the performance of wildtype (WT) and APP23 mice at 12, 18, and 24 months of age in three paradigms of the MWM. Data are expressed by means ± SEM. Day-by-day quantitative analysis of the CUE and PT place leaning task by means of (**A**–**C**) mean escape latency, (**D**–**F**) distance and (**G**–**I**) swimming speed. Open squares or bars: WT mice; Solid squares or bars: APP23 mice. ANOVA, D, day factor; G, genotype factor, Post-hoc Student *t*-test; * *p* < 0.05, ** *p* < 0.01 vs. the corresponding control group. (**J**–**L**) Probe trial for short term memory assessed by means of the time in each quadrant; adjacent right (Ar), Opposite, Target and adjacent left (Al) quadrants. Statistics: Student *t*-test; * *p* < 0.05 vs. the corresponding control group. Target vs. opposite vs. # *p* < 0.05, ## *p* < 0.01.

Figure 5 illustrates the performance of animals in three paradigms of the Morris water maze, namely, the cue, place task, and probe trial. No differences between groups were found in the first CUE task for visual perceptual learning in any of the variables studied. However, a trial-by-trial analysis of their first performance in the maze showed distinct behaviors depending on the age. Thus, both groups performed equally at 12 months of age and tended to differentiate at 18 months and finally did it at a very old age. After that, genotype x day interactions were found in the mean escape latencies in the place task for spatial reference memory, at the three ages studied (Figure 5A–C: G × D*, all F’s [3,45] = 3.30, *p <* 0.05). Thus, the acquisition curves of both genotypes differed through the four days of the test, with a worse performance of APP23 mice as compared to the WT littermates shown as a longer delay to find the platform on the third (PT3, * *p <* 0.05) and fourth (PT4, * *p <* 0.05) day of the test. Genotype differences in swimming speed were shown at 12 months of age (Figure 5G, G**, F [1,15] = 10.79, *p <* 0.01), with a slower pattern in APP23 mice. Therefore, distances covered to reach the platform were also calculated (Figure 5D) but showed a smother distinction in the temporal course between genotypes. At older ages, both groups of mice slowed their navigation speeds, and genotype differences were lost (Figure 5H,I). Statistically significant differences observed in escape latency translated into the variable of distance only at 24 months of age (Figure 5F, PT4, * *p <* 0.05). In the probe trial (Figure 5J–L), independent of genotype, preference for the target vs. opposed quadrant was only shown at 12 and 24 months of age, while in the 18-month-old groups, the time of permanence was biased to the adjacent left quadrant. Surprisingly, the preference of 24-month-old APP23 mice for the target quadrant was significantly higher than their WT littermate counterparts (Figure 5L, * *p <* 0.05).

## 4. Discussion

In the present work, we determined the age and AD-genotype sensitivity of a battery of behavioral tests in APP23 mice of male sex at middle (12 months), old (18 months), and very old (24 months) ages, and compared them to age-matched C57BL/6 mice with normal aging. First, we confirmed that survival curves of APP23 mice were those suitable to assess long-lived animals, since the scarcity of the literature comparing male animals from middle to very old ages is due to increased mortality risk in this sex, increased heterogeneity in the aging process, and also in AD at end-of-life dementia stages.

Genetically engineered AD models, primarily based on the overexpression of presinilin1 or APP mutant human genes causing early-onset Familial AD (FAD), are used to study hallmark behavioral and neuropathological mechanisms of SAD, the sporadic and most common form of Alzheimer’s disease despite some gaps [53,54]. For instance, the APP23 mice, overexpressing the mutant human-type APP protein with the Swedish mutation (K670M/N671L), is a widely used animal model of AD since it genetically develops amyloidosis and presents cognitive and behavioral symptoms similar to those found in patients [55]. However, both SAD and FAD models are limited by the difficulty to fully recapitulate the complexity and progression of this neurodegenerative disease in humans [18,30,54]. Most importantly, this consideration also refers to the challenge to unveil the factors that may underlie the heterogeneity described at end-of-life dementia and the individuals’ survival. The neuro-immuno-endocrine hypothesis could explain the morbidity–mortality paradox among sexes that also accounts for AD [56,57,58], and we have already described it in mice [19,35,46]. However, more recently, distinct genotype and sex differences in life expectancy among different mouse models of AD and inbred strains have been reported in comprehensive reviews [44,45].

Survival—The survival analysis was based on a total of 916 cases from our APP23 and wildtype cohorts with the same gold-standard C57BL/6 genetic background. It showed genotype and sex-dependent increased mortality rates in the mutant mice, with a severe initial drop of survival during the first two months of age, which was more dramatic in APP23 females. Interestingly, APP/PS1 males also exhibit longer lifespans than females [59]. Survival curves demonstrated that APP23 males were able to reach a long life span, rending this animal model suitable for studying the impact of the disease in a male aging scenario. That is, 12- 18- and 24-month-old subjects would serve to study the disease at the middle, old, and very old ages, respectively, and as compared to age-matched C57BL/6 wildtype mice. There is a chance that it is strongly restricted in other genetically engineered AD models where mortality rates of males are a limiting point to address advanced aging in AD [17,22]. As mentioned before, the study of the age-matched wildtype mice also contributed to provide data to the scarcely reported normal aging process in the gold-standard C57BL/6 mice strain, from middle, old, and very-old ages.

Factorial effects and battery of tests—In this research, the behavioral profiles of APP23 and WT mice with normal aging were evaluated through a battery of seven tests. Namely, the corner test, open field test, the novel object recognition test, the T-maze, and three paradigms in the Morris water maze determine the effects of genotype and aging process on their motor, emotional, and cognitive functions. On a general basis, aging was shown as the most determinant factor for most behavioral variables studied, achieving the strongest statistically significant values, whereas genotype factor was specific of those variables related to dysfunctional impairment in AD. This is in agreement with previous results reported by our laboratory using another animal model of AD. There, the age factor from adulthood to maturity (7 to 11 months of age) was also a determinant for variables related to sensory and motor function, while genotype differences specifically pointed at cognitive and BPSD-hallmarks of disease [51]. After the mean life span, the factor of age was shadowing the genetic differences, and a mortality bias was also noted [17,22]. In the present work, the great majority of the variables that were found to be age-sensitive belong to the corner test and the open field, in agreement with the worsening of fear and anxiety-like symptoms with the progress of the disease. Here, genotype differences selectively pointed at hyperactive horizontal activity patterns, in decrement of vertical exploration, resembling those described in 3xTg-AD mice [18].

Corner and open-field tests—Fear and anxiety-like behaviors are the most common neuropsychiatric-like alterations associated with dementia that can be studied in mice models of AD [18] since they appear in their early stages, worsen with the progress of the disease, and most standard behavioral tests can record their presence [60]. Therefore, the battery of tests started with the corner and open-field tests to record the quantitative and qualitative features of these behaviors elicited in these two anxiogenic enclosures. When the APP23 mice were introduced in a new home cage of transference to the open field, the mild neophobia recorded was increased compared to that exhibited by WT littermates and worsened with age. Thus, the mutant mice performed a greater number of visited corners, and the reduction of vertical activity at 24 months of age was more severe, unveiling genotype differences in this variable.

This response was in agreement with the subsequent behavioral sequence of events observed in the open-field, illustrated as an ethogram likeminded with the sequence of ‘action programs’ described by Lát in 1973 [61]. The APP23 animals showed increased latencies to develop the ethogram immediately after direct exposure to the open and illuminated arena. They differed in the momentary freezing, with a longer fear response when confronting the new environment. Furthermore, they differed in the flight-to-fight copying with a stress strategy developed that elicited the early presence of bizarre vertical behaviors (stereotyped rearing without walk support, as described in [51] and anticipatory self-grooming indicative of increased anxiety [18]). With aging, the overall activity and the thigmotaxis behavior showed a reduction in all the groups. This effect was more notorious in the mutant mice. Thus, while 12-month-old APP23 mice were more active than their WT littermates in all (the central and peripheral) areas of the field, the activity of 18- and 24-month-old APP23 mice was strongly reduced in the center, indicating a worsening of anxiety in the elder ages/stages of the disease. In contrast, the increased allocentric exploration close to the protected areas of the mutants was persistent from middle-age until the oldest age. Age and genotype differences were lost the next day, as assessed in the open-field retest. When confronting the open field for the first time, the hyperactive pattern shown is consistent with previous studies describing hyperactivity in APP23 mice when assessed in this test and a Y-labyrinth [62]. However, other laboratories have reported a hypoactive pattern in this animal model [63]. The discrepancies may correspond to the specific anxiogenic conditions elicited by the tests, similarly to hyperactivity of 3xTg-AD mice when assessed under soft light conditions in activity cages. In contrast, they exhibited a clear anxiogenic hypoactive pattern in the open and illuminated open field, the dark lightbox, and the elevated T-maze [17,18].

Gait analysis of locomotor activity—The quantitative and qualitative assessment of locomotion, with distinction of inactivity, and slow and fast movements, allows discriminating patterns of hyperactivity [64]. Here, based on that, the analysis of locomotion measured the walking speed (crossings per interval of time) and its time course through the 5 min of the test [18] and contrasted is to the classical total counts. For the first time, in the present work, we propose measuring two more features of ambulation: the pace (mean distance covered in each walk) and the number of walking episodes. These variables allowed distinguishing the locomotor performance of APP23 mice from the normal gait phenotype of their WT littermate counterparts. Thus, the mean walking distance of a pace during the different minutes of the open-field test was about three–four crossings. This agrees with the number of crossings the animal needs to cover to move from one corner to the closest one, or to cross the arena without reaching the center. Here, all through the test, 24-months-old APP23 performed longer walking distances than their age-matched counterparts and the other groups, probably due to their lower activity in the center. It is important to note that the increased total activity of APP23 mice at 12 and 18 months of age did not result from a walking performance covering a greater number of crossings per pace, but due to a higher number of paces, walking episodes per interval of time.

On the other hand, in the three mutant groups, the total paces per interval of time were also increased at several time points of their temporal courses, and the value trend decreased with age. This variable related to kinetics was more sensitive to discriminate mutants at 12 months of age from their age-matched counterparts. The homologous, the swimming speed in the Morris water maze, also allowed unveiling genotype differences at 12 months of age, though in that case, APP23 mice were swimming slower than controls. Interestingly, longitudinal population-based studies have shown a decline in spontaneous or so-called preferred walking speed (PWS) in older people associated with brain structural changes in gray and white matter volumes [65]. At the clinical level, a slower fast-walking speed (FWS) is used as a marker of frailty and mortality [66,67,68,69]. Besides, the slowest PWS and FWS, and a smaller walking speed reserve (the difference between both) have been associated with poorer cognitive stage [70]. Furthermore, recently, an international cross-sectional gait and Alzheimer interaction tracking study has consistently described an abnormal gait phenotype since the earliest stages of dementia [71]. The Spatio-temporal gait parameters are worse in woman patients and at advanced/severe stages of dementia, although they seem more relevant to non-amnestic forms of cognitive impairment and non-AD dementias [70].

Cognitive function: Habituation and Recognition tests—In the present work, the hyperactivity pattern of APP23 was consistently apparent from middle to very old ages. Hyperactivity can reflect an altered ability to habituate [72], as shown in the psychostimulant effects of caffeine [73] and A_1_R knockout mutant mice [74]. In fact, habituation is considered as a form of rudimentary learning and memory process for recent events [61], and where an animal learns to ignore stimulus with no predictive value [74]. Several other aspects of the cognitive profile were analyzed during the following days through different paradigms.

First, we wanted to assess the cognitive function involved in other recognition tasks. As we have previously shown, the performance of young and adult 3xTg-AD animals in the first minute of a repeated open-field test is sensitive to AD-genotype [52]. In fact, the re-exposure to a fearful environment is the basis of many conditioned memory tests (i.e., [75]). Therefore, on the second day of tests, we assessed the recognition of the open and illuminated field and, after that, the preference for a novel object from one previously inspected was recognized as familiar. The performance on the first minute of the open field repeated test closely resembled the neophobia response previously shown by the same animals in the corner test. Animals maintained their locomotor activity in the periphery and slightly increased the one performed in the center. Overall, that resulted in the loss of genotype differences previously found on the first exposure to the open field. Concerning the novel object recognition test per se, no significant differences were detected between any of the groups as well. The most found was a genotype difference in 24-months-old APP23 mice, with worse latency to explore the novel object than the familiar, but this was due to a better performance in the WT mice. The lack of AD sensitivity of these recognition tasks regarding their use to assess learning and memory could be related to the animal’s anxiety-like phenotype and/or a reduced interest for exploration, the two main potential confounds that can bias the animal’s cognitive performance. This would agree with the genotype and age-dependent reduction of vertical exploratory activity already shown on the first experience in the open field test and the hyperactive horizontal locomotor pattern.

T-maze—Whereas the open field allowed us to observe significant differences associated with age, and to a lesser degree with the genotype, the assessment of animals in the T-maze provided the most AD-sensitive variables. This T-shaped labyrinth is a scenario mainly used to evaluate spatial working memory [76] with the involvement of the hippocampus, septum, prefrontal cortex, and the basal forebrain [77,78,79]. In addition, the latency to reach the intersection of the maze differs according to coping-with-stress strategies expressed by the animal. This is to the extent that this variable was used for psychological selection of prematurely accelerated aging mice (PAM) and non-PAM mice after demonstrating that it correlates with a worse neuro-immunoendocrine function, indicators of accelerated aging, and premature death [80]. We have also extensively shown that the spontaneous alternation in the black corridors resembling burrows is able to unveil the nuances of cognitive function and anxious-like profiles in both male and female mice, from young to old age under healthy and diverse neuropathological conditions [81,82,83]. More recently, some authors have dissected other cognitive aspects [84].

Since the beginning of the test, the ethogram or sequence of behavioral events exhibited by APP23 mice in this maze was strongly affected by genotype. Thus, from a ‘facing the wall’ starting position, the latencies to turn, reach the T-intersection, and cross it with the four paws were significantly delayed in the mutants. The mean time to explore the two arms was increased with age and the AD-genotype. However, this delay was not due to time invested in the vertical exploratory activity, as it was poor and worse with the AD-genotype already since middle age. As a result, APP23 mice took a longer to complete the test as compared to their age-matched wildtype mice, a variable that exhibited a clear and robust genotype effect too. The contribution of errors re-entering an already visited arm was also scarce, with 18- and 24-months-old groups performing slightly better than middle-aged groups. At these ages, there was a trend of worse performance in APP23 mice. In other AD models, such as the 3xTg-AD mice, no differences in the number of execution errors were also found at middle age, but the efficiency to complete the different phases of the test is diminished in 3xTg-AD mice and slightly is affected by long-term treatment with the antipsychotic risperidone [17].

The present results define an altered ethogram in the T-maze and point at the delay to cross the intersection as the most AD-sensitive variable. This variable suggests a worse decision-making process, mainly depending on the prefrontal cortex, which could result from this model’s extensive cortical neuropathology. Compared, the delays in the performance of the 3xTg-AD mice are also found to turn and reach the intersection of the T-maze, but once there, the delay in crossing is not so enhanced, as shown here for the APP23 mice. However, a similar delay suggesting chances in risk assessment is seen in this model when the spontaneous choice of entering an anxiogenic white and lit enclosure is assessed in the dark–light box [18,48,60]. Interestingly, that variable was improved by early postnatal handling, an early life intervention based on a tactile sensorial stimulation experience known to modulate cognitive and anxiety-like behaviors in rodents [51]. The APP/PS1 [85] and Tg2576 [86] mice, as well as selective dopaminergic receptors, cause a blockade in the pre-limbic region of the prefrontal cortex [86] or STOP-null mice modeling schizophrenia [87], also show a reduction of spontaneous alternation, suggesting its relation to frontal hypofunctionality. In the present work, the correlation analysis of the variables of T-maze with other tests indicated that the performance in the maze was strongly related to the variables of the other tests referring to increased emotivity/anxiety (self-grooming, navigation speeds) and apathy (latency of vertical and object exploration).

Cognitive function: Morris water maze—Finally, in the Morris Water maze, short- and long-term spatial reference learning and memory were assessed [88]. The performances of animals in two learning and memory tasks administered during five consecutive days were evaluated through the classical mean escape latency and the distance covered to reach the platform since a genotype effect was found in the swimming speed at 12 months of age. All animals performed equally in the cued learning, an easy task that assesses visual perceptual learning. Still, it is interesting to note that the performance in their first experience in the maze differed with aging, as very old APP23 confronted the stressful situation of being immersed in a water tank with faster latencies and shorter distances than their WT littermate counterparts. As mentioned before, the navigation was slower in APP23 at middle age but not after that. Here, the impact of aging could explain that the swimming speed be reduced in old and very old animals as compared to the younger groups. If so, the slow swimming speeds of middle-aged APP23 mice compared to their WT littermate group but similar to the speed shown at 18 and 24 months of age, would suggest that they were performing as older than expected for their chronological age. Although the anxious profile of 3xTg-AD mice explains their faster swimming speeds from early stages of the disease [89], we have previously shown a similar situation regarding AD-behavioral performances being similar to those corresponding to WT at older ages, both at the biological [90] and behavioral level [19]. In contrast, the genotype differences become strikingly smoother in long-term AD mice survivors due to a mortality bias (death of the worse animals) on one hand and age-dependent impoverished performances in the WT groups on the other [22]. Here, the performances of 24-month-old animals were not as bad as could be expected in a progression from 18 months of age, whereas cognitive impairment was a salient behavior and followed the expected deterioration.

After the removal of the platform, the performance of animals did not indicate differences in short-term memory per se. This lack of genotype differences in the probe trial two hours after the last trial of the place task was surprising as expected from the worse performance of mutants in the last or two last days of this task that involves the contribution of long-term but also short-term learning and memory. However, it is noteworthy to mention that the lack of sensitivity of probe trial to the genotype in APP23 mice is in agreement with previous results reported by other laboratory experts in this animal model [55,91]. On the other hand, we have recently demonstrated a mortality bias, with animals that died during the behavioral assessments or those with worse life prognostics who had been excluded from the final analysis, explaining the lack of expected results in 16-month-old 3xTg-AD males [17]. However, cognitive deficits in the Morris water maze were the salient behavior in 18-month-old survivor females despite a flat phenotype, not distinguishable from their control strain [22]. Here, the distinct preferences for the adjacent areas indicate, for instance, less-focused goal-directed swimming strategies at 18 months of age [91]. This suggests that a swimming strategy analysis would further understand the compensatory behavioral mechanism that resulted in such a paradox probe trial report [91].

Limitations—The main limitation of the present study is intrinsic to the heterogeneity of the aging process and the extreme difficulties to obtain the desired sample size of old and very old animals, not only of mutants with such high mortality rates, but also of their wildtype littermates with normal aging. In order to counteract the age-related heterogeneity, important efforts were needed to obtain concurrent middle-aged (12-month-old), aged (18-month-old) and very old (24-month-old) animals with the APP23 and WT genotypes, so they shared the same living conditions; moreover, to adapt research agendas so that the cohorts could be assessed within a narrow frame of time in the calendar. On the other hand, as discussed, the use of a battery of tests must assume that carry-over effects cannot be discarded, despite the benefits that assessing the same construct (i.e., anxiety) in tests differing in the level of anxiogenic conditions has to ensure convergent validity of results. These efforts resulted in a low intra- and inter-group variability, and we have reproduced the findings in subsequent experimental research in a consistent manner. However, interlaboratory reliability would be needed to confirm that the results obtained in our colonies can be reproduced in the colonies of other research institutions. An important limitation would refer to gender medicine demands for the concurrent comparative study of males and females, so future efforts should be devoted to achieve this goal despite the difficulty that it may represent when handling very old ages and survivors.

## 5. Conclusions and Future Directions

In the present work, the behavioral profiles at 12, 18, and 24 months of age of male APP23 mice and non-transgenic (WT C57BL/6) littermate counterparts with normal aging were assessed using three independent cohorts but in a narrow temporal frame in the calendar. Animals were successively evaluated in a battery of seven behavioral tests to screen motor, non-cognitive and cognitive-like symptoms comprehensively. Presence of ‘Behavioral and Psychological Symptoms of Dementia’ (BPSD)–like behaviors in APP23 mice was confirmed in three classical unconditioned tests evaluating locomotion, exploration, anxiety-like behaviors, and emotionality under three different anxiogenic conditions. 

The main findings can be summarized in five points as follows:

(1). Survival curves of 920 mice of APP23 and WT C57BL/6 littermates confirmed genotype and sex-dependent increased mortality rates, and long-living males.
(2). Compared to WT littermates, the APP23 mice showed an increased number of visited corners but decreased vertical exploratory activity, evidencing increased neophobia in the corner test. Similarly, APP23 groups showed increased latencies to develop the ethogram of behaviors immediately after the direct exposure to an open and illuminated field (not shown) and increased locomotion, with increased thigmotaxis (or search for the protected peripheral area), primarily noticeable at 12 months of age.
(3). In the T-maze, a black T-shaped corridor resembling burrows, the latency to reach the intersection was consistently shown to be increased at all ages and the longer time required for APP23 mice to complete the exploration of all the arms of the maze. As part of the differences in the status of their cognitive functions exhibited during the exploration of new environments, we assessed APP23 mice for putative cognitive deficits in discriminative tasks (novelty/familiarity) for context (remembering the anxiogenic open field scenario) and object recognition.
(4). The novel object recognition test showed low sensitivity to the genotype, only noticed at the oldest age through the lowest difference in the delay in exploring the novel and familiar objects compared to WT littermates.
(5). Cognitive deficits in Spatial reference learning and memory were assessed in the Morris water maze through a 4-day place learning task using a hidden platform followed by a probe trial for long-term memory where the platform had been removed. Worse performance of 12-month-old APP23 mice was shown as compared to WT littermates during the progress of acquisition (learning and memory) in the place task, while differences were scarce in the older groups. In the probe trial, the worse spatial memory discrimination of the trained quadrant compared to the opposed quadrant was shown at 18 months of age but independently of the genotype. As we have reported in other mice models for AD, a conspicuous BPSD-like profile and age as a biological factor may result in confounding factors in an aquatic maze, considered an anxiogenic environment for mice.

In summary, most of the variables analyzed were able to show age-related differences, and those presented here are among those that, under our experimental conditions and in our hands, better discriminate the behavioral phenotype of female APP23 mice through their aging process, from middle-age (12 months of age) and old (18 months of age) to very old (24 months of age), as compared to their respective WT littermates. Most importantly, non-linear age- and genotype-dependent behavioral signatures were found in 24-month-old mice, suggesting the existence of a crosstalk between chronological and biological/behavioral ages in long-lived animals useful to study underlying mechanisms and distinct compensations through natural physiological aging and, maybe also, long-term AD-associated aging male survivors.

## Figures and Tables

**Table 1:** Genotype and age factors and interaction effects on behavioral tests and variables from middle-age to long-life.

Behavioral Tests and Variables	Statistics	Genotype	Age	G × A	Significance
Corner test					
Total visited corners	F (2,52) = 7.392	-	A ***	-	*p* = 0.001
	F (2,52) = 4.126	G *	-	-	*p* = 0.047
Total numbers of rearings	F (2,52) = 13.374	-	A ***	-	*p* = 0.000
Latency of rearing (s)	F (2,52) = 10.210	-	A ***	-	*p* = 0.000
Open field test					
Freezing—Latency of first movement (s)	F (2,52) = 13.362	-	A ***	-	*p* = 0.000
	F (2,52) = 3.664	-	-	G × A *	*p* = 0.032
Latency to exit the center (s)	F (2,52) = 6.640	-	A **	-	*p* = 0.003
	F (2,52) = 11.169	G **	-	-	*p* = 0.002
Latency of rearing (s)	F (2,52) = 2.905	-	-	G × A (*)	*p* = 0.064
	F (2,52) = 2.649	-	A (*)	-	*p* = 0.080
Latency of self-grooming (s)	F (2,52) = 5.917	G *	-	-	*p* = 0.018
Total horizontal activity (*n* crossings)	F (2,52) = 12.909	-	A ***	-	*p* = 0.000
	F (2,52) = 6.043	G*	-	-	*p* = 0.017
“in the center (*n* crossings)	F (2,52) = 3.044	-	-	G × A (*)	*p* = 0.056
“in the periphery (*n* crossings)	F (2,52) = 11.655	-	A ***	-	*p* = 0.000
	F (2,52) = 9.404	G **	-	-	*p* = 0.003
Total vertical activity (*n* of rearings)	F (2,52) = 18.966	-	A ***	-	*p* = 0.000
Context and object recognition tests					
Latency to enter into the periphery	F(2,52) = 13.448	-	A ***	-	*p* = 0.000
Total horizontal activity					
“in the center (*n* of crossings)	F(2,52) = 3.325	-	A *	-	*p* = 0.044
“in the periphery (*n* of crossings)	F(2,52) = 3.339	-	A *	-	*p* = 0.043
Time exploring new object (s)	F(2,52) = 3.746	-	A *	-	*p* = 0.030
T-maze					
Latency to cross the intersection (s)	F(2,51) = 27.978	G ***	-	-	*p* = 0.000
Total time exploring both arms (s)	F(2,51) = 6.005	-	A **	-	*p* = 0.005
	F(2,51) = 4.107	G *	-	-	*p* = 0.048
Total time to complete the test (s)	F(2,51) = 19.484	G ***	-	-	*p* = 0.000
Total number of errors (*n*)	F(2,51) = 3.139	-	A (*)	-	*p* = 0.052
Total vertical activity (*n* of rearings)	F(2,51) = 9.700	G **	-	-	*p* = 0.003
	F(2,51) = 3.631	-	-	G × A *	*p* = 0.034

Statistical analysis: Factorial analysis, G, genotype, A, age, * *p* < 0.05, ** *p* < 0.01, *** *p* < 0.001; (*) one-tailed.

## Data Availability

The data presented in this study are available on request from the corresponding author.
